# Idiopathic red ear syndrome: A rare case report

**DOI:** 10.1002/ccr3.5564

**Published:** 2022-03-11

**Authors:** Brihaspati Sigdel, Amrit Pokhrel

**Affiliations:** ^1^ Department of Otolaryngology & Head and Neck Surgery Gandaki Medical College Pokhara Nepal; ^2^ Metrocity Hospital Pokhara Nepal; ^3^ Department of Emergency Metrocity Hospital Pokhara Nepal

**Keywords:** dietary trigger, lifestyle modification, migraine, red ear syndrome, trigeminal neuralgia

## Abstract

Red ear syndrome (RES) is a very rare disorder that is characterized by a unilateral or bilateral attack of paroxysmal burning sensation and reddening of the external ear. The duration of symptoms ranges from a few seconds to hours. It can occur spontaneously or be triggered by rubbing of the ear, heat or cold stimulation, brushing of hair, and neck movement. Diagnosis and treatment of this condition are challenging. The pathophysiology of RES is still unclear and hypotheses involving peripheral or central nervous system mechanisms have been proposed. RES is regarded as refractory to medical treatments, although some migraine preventative treatments have shown moderate benefit mainly in patients with migraine‐related attacks. We report a case with Idiopathic RES who presented with paroxysmal redness of the bilateral pinnae partially benefitted by medical treatment.

## Graphical abstract

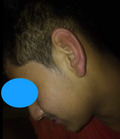

## 1 INTRODUCTION

Red ear syndrome (RES) is a rare clinical entity that frequently does doctors shopping for treatment. The patient of RES presents with a history of paroxysmal erythema of one or both ears with a burning sensation or earache. The onset of symptoms could be either spontaneous or triggered by touch, stress, coughing, sneezing, neck movements, chewing, and combing hair.^1^ Lance described red ear syndrome in 1994 where he studied 12 cases of RES with intermittent assaults of one‐sided ear uneasiness or burning related with erythema of the ipsilateral ear.^2^ Primary form RES is associated with migraine and trigeminal autonomic cephalalgias (TACs), whereas secondary RES is associated with upper cervical spine syndrome, cervical arachnoiditis or spondylosis, traction injury of upper cervical roots (UCR), atypical glossopharyngeal or trigeminal neuralgias, temporomandibular joint (TMJ) dysfunction, or thalamic syndrome.^3^ Raieli et al. found that about 20% of patients with migraine had RES during migraine attack. The duration of attack was less than one hour in 35% of cases. However, in 50% of cases, the duration of attack is unknown.^4^ Symptomatic management and avoidance of triggers should be routinely done for management. Here, we report a 14‐year‐old boy of idiopathic RES who presented to our OPD tired of visiting several doctors in the town since 2 years of onset of the disease.

## 2 CASE PRESENTATION

A 14‐year‐old boy presented to the otorhinolaryngology OPD of Metrocity Hospital with complaints of episodic erythema of both the ears for 2 years. Patient used to suffer about 2–3 attacks of such episode every week. Each episode used to last about 30 to 45 minutes no relieving factors. In further history, patients said that the erythema of the ears was associated with a burning sensation which corresponded to 5–6 out of 10 on visual analog scale (VAS). Furthermore, the patient reported that the scenario was aggravated after awakening from sleep and occurs mainly in summer season. However, the patient did not give any history of earache, headache, blurring of vision, ear discharge, ear fullness, blurring of vision, ringing sensation in‐ear, or difficulty in walking, or burning sensation in hands and feet.

**FIGURE 1:**
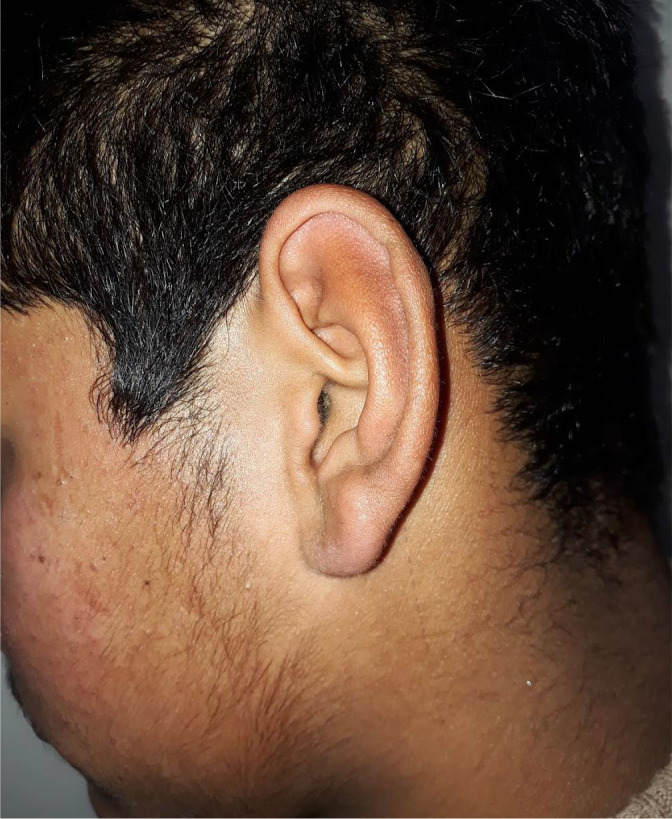
Showing normal‐looking left pinnae

**FIGURE 2:**
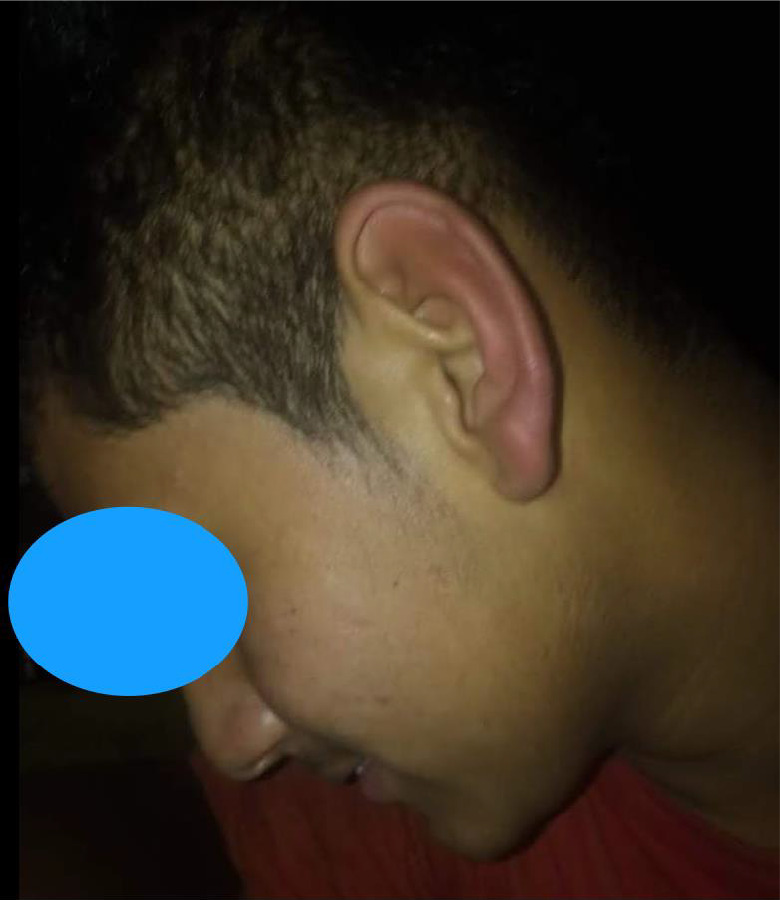
Showing erythematous left pinnae suggestive of red ear syndrome

**FIGURE 3:**
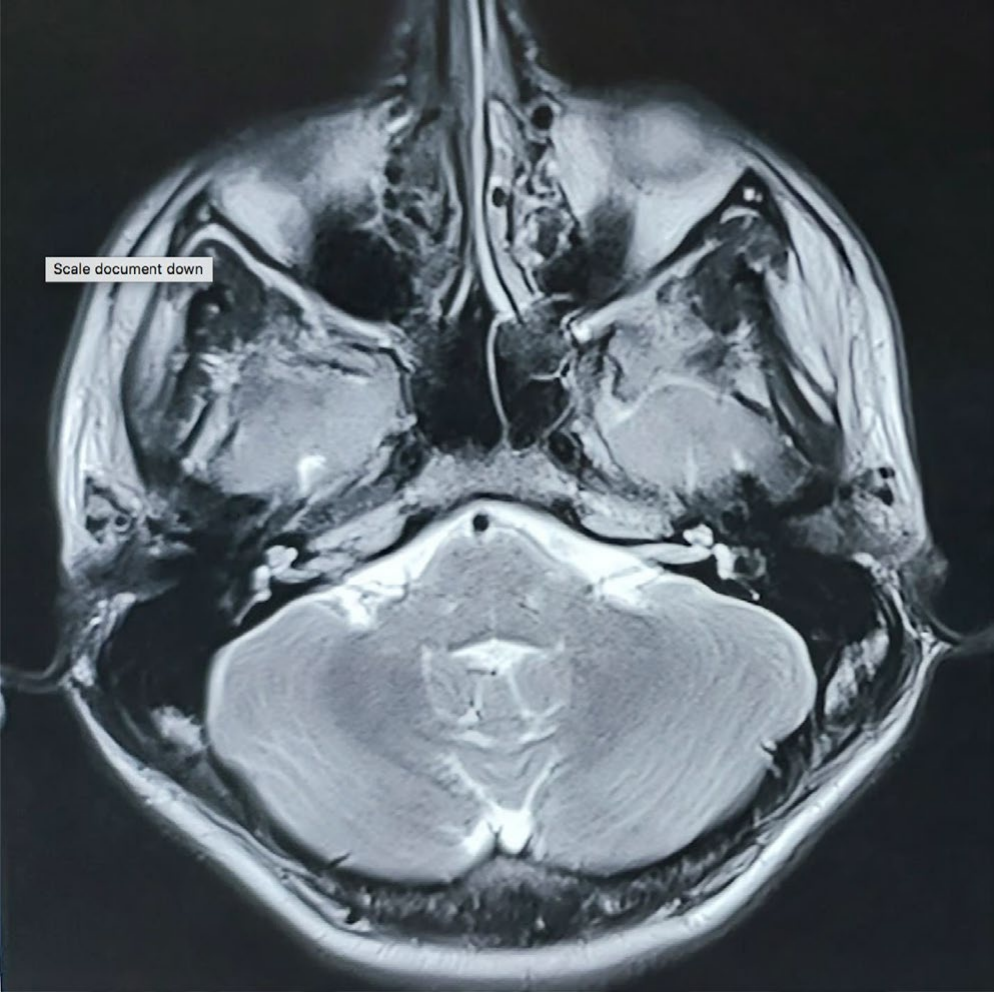
T2 MRI brain showing normal finding

On examination, the patient was well oriented to time, place, and person. Examination of Ear revealed normal‐looking pinna. Otoscopic examination of both the ears was normal. (Figure 1) Neurological examination of the patient was normal. Furthermore, the patient showed the picture on his mobile which revealed bilateral erythematous ear aggravated by sleep. (Figure 2) He has been taking amitriptyline and methylcobalamin tablets for 2 years. His thyroid function tests and blood sugar levels were normal. Magnetic Resonance Imaging of brain was normal (Figure 3). However, the patient was not satisfactory as he was not relieved of the symptoms.

We made the diagnosis of red ear syndrome and prescribed him tablet indomethacin 25 milligram tablets three times a day for 14 days. The patient was advised to follow‐up after 14 days and to report immediately if he suffers from a similar attack of the erythematous red ear. However, after 7 days, the patient did not complain of any such episodes in 14 days. The patient was kept on regular follow‐up for the next 2 months. During this period, the patient said that he had partial improvement compared with the past in the form of a decrease in the number of attacks about 3 per month and severity of pain about 3–4 out of 10 on VAS.

## 3 DISCUSSION

Red ear syndrome is a rare diagnosis. It is a diagnosis of exclusion. It was first described by Lance in 1994. The criteria for the diagnosis of red ear syndrome as proposed by Lambru in 2013 include 20 attacks of erythematous ear lasting for four hours with an earache. These attacks usually occur more than once a day and have at least two of the following features: burning pain, unilateral, mild to moderate severity, and triggered by thermal or cutaneous stimulation of the ear. ^3^, ^5^ The exact cause of red ear syndrome is not yet known.

However, several trigger factors are found in some patients with red ear syndrome‐like touch, exertion, heat or cold, stress, and even dietary factors.^1^, ^6^ Lance reported that this pathology is commonly induced in patients with cervical nerve root disorder, with a predominance of C3 root discharge leading to the antidromic release of vasodilator peptides. Furthermore, he proposed that the primary mechanism of RES is due to activation of the trigemino‐autonomic circuit. As per him, red ear syndrome must be primarily due to the enhancement of sympathetic vasodilation as the ear has less parasympathetic vasodilation than in the nose and cheeks. In our case, the trigger factor was altered sleep and the hot climate. Furthermore, in our case, the patient denied the history of burning sensation in hands and feet which helped us to rule out erythromelalgia in our patient. Other major differential diagnosis is relapsing polychondritis. This rare condition can be differentiated from red ear syndrome as episodes would be expected to last longer than red ear syndrome and relapsing polychondritis clinically would spare the lobule of the ear as the lobule does not contain cartilage, whereas red ear syndrome would usually involve the whole pinna including the lobule as in this case.^7^

There are several treatments proposed in the management of red ear syndrome. However, the exact treatment for the cure is yet unknown. Some of the medications used for the treatment of red ear syndrome are amitriptyline, flunarizine, ibuprofen, indomethacin, verapamil, imipramine, methysergide, and many more. Our patient was responsive to indomethacin. Chance et al reported a similar response observed with indomethacin.^8^

## 4 CONCLUSION

Idiopathic red ear syndrome is a rare diagnosis of exclusion. Symptoms include reddening of the ear aggravated by several factors like awakening from sleep, especially at hot weather,. This pathophysiology is due to parasympathetic vasodilation. It is a therapeutic challenge for doctors. Intracranial and extracranial pathology has to be ruled out before a diagnosis of red ear syndrome is made. Clinical suspicion is needed to improve the quality of life.

## CONFLICT OF INTEREST

None.

## AUTHOR CONTRIBUTIONS

BS conceptualized the study. BS and AP involved in design, literature review, writing and approving the final manuscript.

## CONSENT

Written informed consent was obtained from both the patient and his parents for publication of this case report and any accompanying images.

## Data Availability

Data available on request from the authors.
